# Predicting Inpatient Length of Stay in Iranian Hospital: Conceptualization and Validation

**DOI:** 10.31557/APJCP.2020.21.8.2439

**Published:** 2020-08

**Authors:** Omid Khosravizadeh, Soudabeh Vatankhah, Mina Jahanpour, Negar Yousefzadeh, Saeed Shahsavari, Saeed Yari

**Affiliations:** 1 *Social Determinants of Health Research Center, Research Institute for Prevention of Non-Communicable Diseases, Qazvin University of Medical Sciences, Qazvin, Iran. *; 2 *Department of Health Services Management, School of Health Management and Information Sciences, Iran University of Medical Sciences, Tehran, Iran. *; 3 *Department of Health Services Management, School of Management and Medical Information Sciences, Isfahan University of Medical Sciences, Isfahan, Iran.*; 4 *Health Management and Economics Research Center, Iran University of Medical Sciences, Tehran, Iran. *; 5 *Health Products Safety Research Center, Qazvin University of Medical Sciences, Qazvin, Iran. *; 6 *Department of Epidemiology and Biostatistics, School of Public Health, Tehran University of Medical Sciences, Tehran, Iran. *; 7 *Student Research Committee, (Department and Faculty of Health), Shahid Beheshti University of Medical Sciences, Tehran, Iran. *

**Keywords:** Predicting, inpatient length of stay, hospital, Iran

## Abstract

**Objective:**

The length of stay is an important indicator of hospital performance and efficiency. Regarding the importance of the length of stay, this study aimed to design a structural model of the inpatients’ length of stay in the educational and therapeutic health care facilities of Iran in order to identify the influencing dimensions.

**Methods:**

The present study was an analytical and applied study. The face validity of the data gathering tool was investigated by the expert judgment and the construct validity was examined by using the exploratory factor analysis. In order to verify the reliability of the tool, the internal consistency was also trialed by using the Cronbach’s alpha. For ranking the influencing dimensions and factors and also in order to examine the causal relationships between the variables in a coherent manner and presenting the final model, the structural equation modeling technique was used in AMOS software at a significant level of 0.05.

**Results:**

The mentioned structural model consists of 4 dimensions and 29 factors influencing the length of stay of hospitalized patients. The independent variables are based on priority and importance as follows: patients’ conditions, the underlying factors, the clinical staff performance, and hospitals’ service delivery, which were examined by second-order factor analysis in order to study the relationship between them and the inpatients’ length of stay.

**Conclusion:**

Considering the importance of each one of the proposed dimensions from the point of view of service providers in some therapeutic centers of the country by paying attention to the role of each one of them in preventing prolonged hospitalization can be essential in the effectiveness of the treatment and cost reduction.

## Introduction

Hospital is one of the components of the health care system, that its’ performance can lead to the health of the community in coordination with other factors. Actually, hospitals have a key role in providing health services because of their impact on the health system’s efficiency (Khosravizadeh et al., 2016). In this regard, in the health care system, a kind of management can be productive which provides high quality and cost-effective services (Mohebbifar et al., 2014), achieving this goal will be possible by the correct and reasonable use of resources, controlling hospital admissions and the length of stay of hospitals patients and also the appropriate use of diagnostic and therapeutic services (Ravangard et al., 2010). The length of hospital stay is often considered as a measure and an indicator of the efficiency and effectiveness of hospital services, as well as the effectiveness of used treatments by the physicians (Austin et al., 2002). The presence of indices such as bed occupancy rates and bed turnover or bed turnover interval make the length of stay one of the most important functional indicators of the hospital (Gohari et al., 2012). In fact, the length of stay is defined as the time between admission and discharge in the hospital, which measures the rate of bed use and the efficiency of the admissions (Jimenez et al., 1999). Therefore, the mentioned performance indicator is dependent on the health care delivery variables, including the availability of hospital beds, payment methods, and hospital discharge policies, as well as the variables of the demand for health services, including the severity of illness, direct and indirect costs and simultaneous illnesses (Clarke, 1996). Hence, the length of stay more or less than the actual need of patients can affect the cost and quality of the provided care (Mawajdeh et al., 1997). So, improving the patient’s length of stay not only reduces costs and improves hospital performance, but also reduces the unnecessary beds’ occupancy and increases the hospitals’ productivity (Haghgoshaei et al., 2012). In addition to the fact that the length of stay is a crucial factor in analyzing the performance of both clinical and para-clinical units, it can also be measured as the clinical performance during the comparison of the two or more factors in different units, such as surgery, gynecology, emergency, and pediatrics (Weingarten et al., 1994). Various studies have also shown that length of stay can be affected by several factors such as age, sex, income, status, education, marital status, severity of disease, type of insurance, breed, number of beds, hospital size, the area in which the hospital is located and the type of physician’s activity as a family physician, private physician (in office) or a hospital physician (Mawajdeh et al., 1997; Toyabe et al., 2006). In other studies, some other factors such as birthplace, place of residence, admission time, type of admission, hospitalization history, patient status at the time of discharge, and severity of illness have been mentioned as influencing factors on hospital length of stay (Gholivahidi et al., 2006; Rajaeefard and Rafiee, 2006). Also, findings from a conducted study in some hospitals in Japan indicated that an indirect relationship exists between the length of stay and accessibility to the human resources during the hospitalization and there is a direct relationship between the length of stay and the hospitalization capacity and rate of unnecessary and unwanted admissions(Imai et al., 2005). As noted, studies on the patients’ length of stay have generally reviewed the factors including clinical and non-clinical factors affecting it with a general overview. In the majority of these studies, the variables of the patients’ admission aren’t considered especially in a model. Also, in these studies, a comprehensive model hasn’t presented by considering all the clinical and non-clinical factors affecting the patients’ length of stay. In the current study, a model has been presented with a wider perspective and with consideration of all factors affecting the length of stay. The results can be useful for better planning of services and appropriate decision making in the field of hospital services, especially about the length of stay, the proper use of resources and maximal productivity at the hospital level.

## Materials and Methods

From the point of view of purpose, this study was descriptive-analytic and cross-sectional. Due to the fact that many appropriate solutions and approaches can be presented and applied based on the results of the study, the nature of the research is also applicable. The present study was composed of three phases.

*A comprehensive review of studies*

At this phase, we reviewed the previous studies on the length of stay of hospitalized patients using databases. The tool used at this stage was a data collection form. This form was used in order to gain integrity, reduce bias, and increase the reliability and validity. Required data were collected from various databases. The databases include Irandoc, Embase, Google scholar IranMedex, SID, Magiran, PubMed, Scopus, WOS and Keywords were used such as length of stay, Hospital, patient admission, Affective factors and their equivalent in Persian. Then the articles were reviewed in terms of quality. Then factors influencing the length of stay were classified. Also, the most relevant factors were tried in one category. The proposed basic model of this study is presented. The current model is obtained by systematically analyzing the results of a comprehensive review of studies. In this model, the components of the service delivery, medical personnel, patients, and background are conceptualized as independent dimensions and the length of stay of inpatients is considered as dependent dimensions.

*Designing a tool and data collection*

The questionnaire was compiled from the obtained information from the first phase as well as the opinion of the supervisors and counselors. In this study, the expert judgment was used for evaluating the face validity. Exploratory factor analysis was used to assess the construct validity. Cronbach’s alpha was used to measure the reliability of the questionnaire and its rate was 0.917. The five-section Likert scale was used to determine the importance of factors affecting the length of stay. So that the importance of the factors was categorized from very low, low, medium, high and very high. After collecting the data, the factors influencing the length of stay were ranked. The study population has consisted of all the staffs of the clinical units including physicians, nurses, paramedics and financial experts and clerks of educational and therapeutic centers in Tehran province, and the quadrat sampling was performed for each hospital. Since the size of the study population is unlimited, the following formula was used for determining the sample size. In the following formula, α is the estimated error or estimation error equal to 0.05, and ε is the possible error rate or accuracy required in the survey, which is equal to 0.05. Therefore, taking into account 95% confidence level, the standard deviation of 0.5 and margin of error (+ -5%), the sample size was calculated to be 384 patients. Finally, 390 questionnaires were distributed. Descriptive statistics were also analyzed by using the AMOS software.

*Validation and presentation of the final model*

At this Phase, the final model was presented based on the quantitative findings. In order to study the causal relationships between variables in a coherent way and presenting the final model, the technique of the structural equation model was used. This technique consists of five stages as follows: Model expression: (including constructing the original model), model estimation: Data Collection and Creation of Variables Matrices), Goodness of Fit Assessment: (Including a general review of the model’s appropriateness and its feasibility, and the measurement of the need for reform), model modification, model interpretation. The above steps were done through AMOS software; the final validated model was presented.

## Results

**Figure 1:**
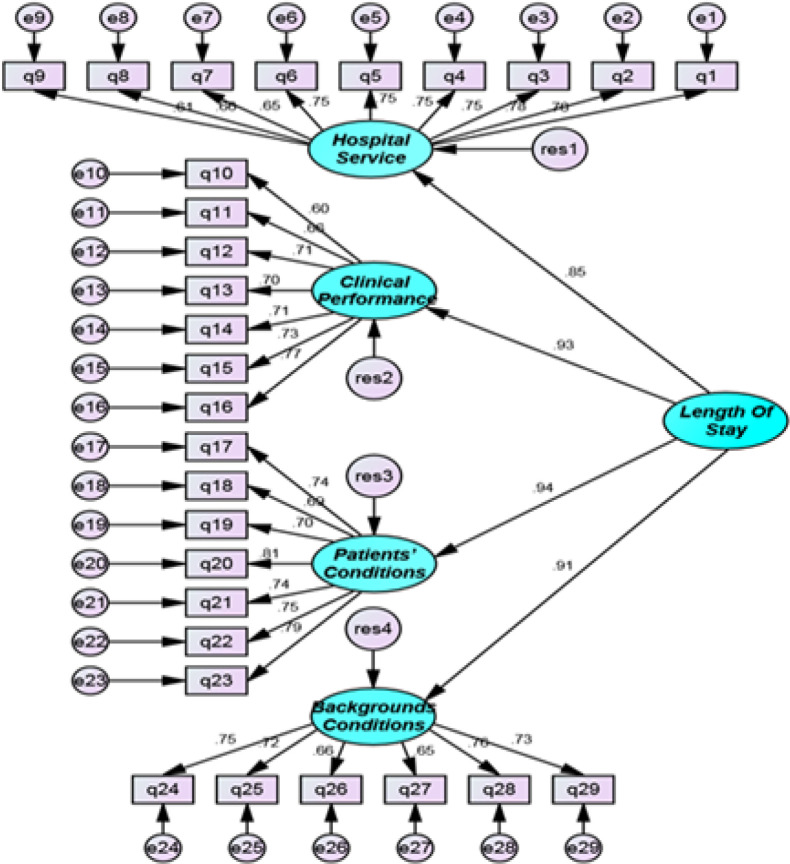
Standard Estimation Coefficients for the Second-Order Factor Analysis of the Variable of the Length of Stay in the Conceptual Model

**Figure 2:**
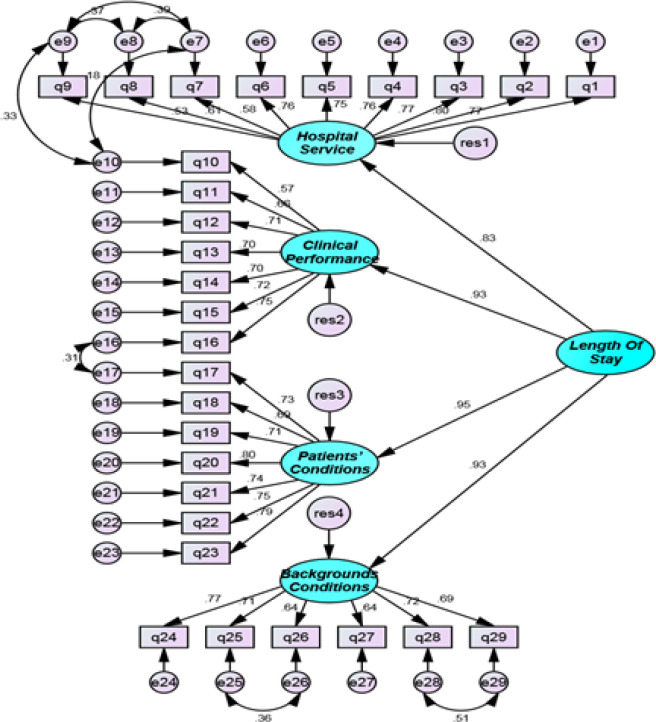
Standard Estimation Coefficients for the Second-Order Factor Analysis of the Variable of the Length of Stay in the First Proposed Model

42.5% of the participants were male and 57.95% of them were female. Most of the respondents were in the age group of 31-40. Also, most of them had bachelor’s degree in nursing. The work experience of the majority of respondents was about 10 to 20 years. Reviewing the findings in the field of the independent variables of the study showed that the highest mean of dimensions related to the patients’ conditions and the lowest mean related to the performance of the care providers in the studied educational and therapeutic centers. To evaluate the compatibility and consistency of the model with the research data, the fitness of the model was verified; the fitness of the conceptual model was studied in two steps. The first step was the assessment of the measurements of the model and the second is the assessment of the fitness of the structural part of the model. In order to assess the measurements of the model, the validity and reliability of the model were evaluated. The second-order factor analysis was performed to determine the relationship between patient’s length of stay and its dimensions. Figure 1 shows the standard estimation coefficients of the second-order factor analysis of the variables of patients’ length of stay. All paths are at the significant level. According to the table 1, the obtained values for the indices such as Qi-2 divided to the degrees of freedom, 1GFI, 2 RMSEA and 3 CFIs weren’t within the defined range. Therefore, it was concluded that the fitness of the model wasn’t appropriate at this phase, so applying some changes to the model were considered necessary for better fitness. These changes were implemented in the proposed model and the results of the fitness indices improved (Figure 2).

Also, the effects of each factor on the mentioned variable were prioritized according to the standard estimation coefficients of the second-order confirmation factor analysis of the patient’s length of stay variable. The significance level of the patient’s length of stay and the influencing factors presented in Table 2. The correlation coefficient between these four factors and the patient’s length of stay indicated the effect of these variables on the length of the patient’s stay. based on the priority and the extent of the effect of these dimensions on the length of stay, the variable of patients with a value of 643.9 which is greater than 1.96 indicated that the relationship between the variable of the patient’s length of stay and the patients is significant at 95% confidence level and ranked as the first important variable among others. Then the variable of the background and variables of the clinical staff and hospital services obtained the most importance and priority.

**Figure 3:**
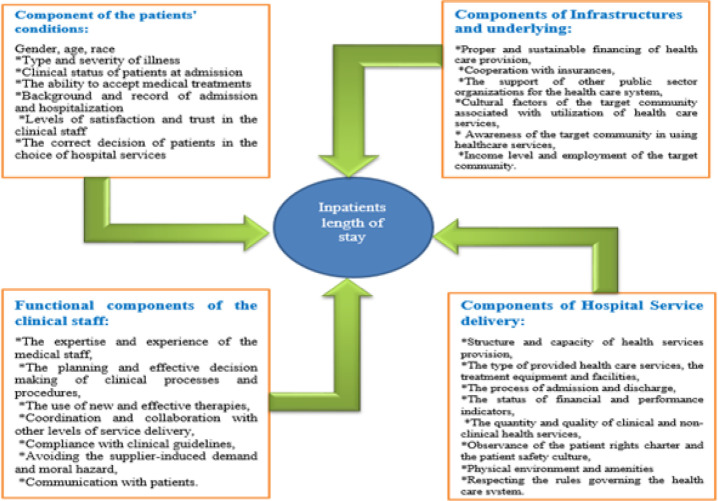
Final and Valid Model

Also, by considering the linear and significant relationships between independent and dependent dimensions, by using the structural equation technique, the final model included 4 independent dimensions and 29 factors influencing the length of stay of inpatients which is presented in Figure 3

**Table 1:** Comparison of Fitness Indices in the Conceptual Model and the Proposed Model 1

fit index	Permissible limit	Conceptual model	Proposed model 1
$\left(\frac{x^{2}}{d_{f}}\right)$	<3	3.88	2.84
GFI	>0.9	0.764	0.838
RMSEA	<0.08	0.086	0.069
CFI	>0.9	0.848	0.905

- Goodness of Fit Index (GFI); - Root Mean Square Error of Approximation (RMSEA);- Comparative Fit Index (CFI)

**Table 2:** The Results of the Second-Order Factor Analysis of the Patients’ Length of Stay in the Final Model

Variable	Dimensions	The Significant number	Path Coefficient	Rank	Standard deviation
Length of Stay	Hospital Services delivery	9.352	0.835	4	0.132
	Clinical staff	9.363	0.926	3	0.153
	Patients conditions	9.643	0.95	1	0.157
	Background & underlying factors	8.919	0.93	2	0.153

## Discussion

Increasing patients’ population, lack of resources and high treatment expenditures attract the hospital administrators and patients’ attention to the length of stay in hospital (Ameri et al., 2015). The length of stay in the hospital is an important indicator that demonstrates the hospital’s performance and efficiency and is influenced by various clinical and non-clinical factors (Xiao et al., 1997). In the present study, these factors are conceptualized and classified into four categories based on their priority and importance, including patients’ condition, background, the performance of the clinical staff, the provision of hospital services and service delivery in Iran.

*Component of the patients’ conditions*

The variable of the patients’ conditions included four elements: gender, age, race; type and severity of illness; clinical status of patients at admission; the ability to accept medical treatments; background and record of admission and hospitalization; levels of satisfaction and trust in the clinical staff; the correct decision of patients in the choice of hospital services. In confirming the conceptualized elements, Deister et al., (2017) also pointed out that the patient’s age determines the length of his stay in a hospital. Therefore, it can be claimed that older patients need more time to improve their disease, because most of them have chronic illnesses, while younger patients are more likely to be affected with acute illnesses, with shorter treatment periods (Lim and Tongkumchum, 2009) Statistics released by the US Department of Statistics revealed that the incidence of various types of diseases is not equal in men and women (Zand et al., 2010), and the length of stay varies according to gender. In confirming the impact of race on the length of stay, in 2017 Turgeman also acknowledged that race has an effect on the length of the patient’s stay (Turgeman et al., 2017). The type and severity of illness are one of those factors affecting the length of stay in the hospital and in line with this conclusion, Paul Carter considered the severity of the illness to be effective over the length of stay in the hospital (Carter et al., 2016). In Japan, Junko Niimura et al. noted that the severity of the illness is one of the predictors of the length of stay of patients (Niimura et al., 2017). The clinical status of patients on admission is one of the factors influencing the length of stay in the hospital. The clinical status of the patient on admission including the patient’s vital signs, physical examination, level of consciousness and background are underlying conditions that determine the length of stay in the hospital (Wuerz et al., 2000). In concordance with the conceptualized element, a study by Carter’s Paul demonstrated that the patient’s clinical status on admission can influence the length of stay (Carter et al., 2016). Liver Turgeman et al. have also emphasized that the patient’s clinical condition on admission was one of the factors influencing the length of stay (Turgeman et al., 2017). Ability to accept a medical treatment means the patient’s informed decision and agreement with the medical advice and treatment procedures with the condition that refusing the treatment can lead to the exacerbation and increase in the severity of a disease or its signs and symptoms that are significantly associated with the prolonged patients’ stay in the hospital. In confirmation of this conceptualized component, Alosco’s study suggested that refusing some therapeutic procedures, especially in patients with heart failure, increases the length of stay in the hospital (Alosco et al., 2014). History of previous admission and hospitalization should be recognized on the admission. Several studies aimed to confirm this conceptualized element, for example, Jordan et al, approved that the history of a disease and background affects the patients’ length of stay (Gruskay et al., 2015). The level of satisfaction and trust in the clinical staff is also an important component and patients are at the heart and main focus of the hospital and all hospital services are performed for them, so their satisfaction is an important indicator of the quality of health care services. In this regard, a study by Kazemi et al., (2010) confirmed that facilities and services of the operating room and other units have the most effect on patients’ satisfaction and there was a significant relationship between the length of stay and total patient satisfaction. Correct and specific patients’ decision making in choosing health care services is also important and granting him/her the right to decide on his or her health along with providing training and necessary information for the correct actions is one of the basic principles of the patient support. Research and experience have shown that informing the patients and contributing them in decision-making in relation to their treatment and respecting their rights improve their health status and wellbeing and also reduce the patient’s length of stay in the hospital (Leenen, 1996). Studies in this area demonstrated that knowledge of the services influences on the patients’ decision making and can improve the effectiveness of the therapeutic processes (Khosravizadeh et al., 2017).

*Components of Infrastructures and underlying*

 The Infrastructures and underlying elements include the proper and sustainable financing of health care provision, cooperation with insurances, the support of other public sector organizations for the health care system, cultural factors of the target community associated with utilization of health care services, awareness of the target community in using healthcare services, income level and employment of the target community. On the whole, proper financing of health care services has a unique role, and financing and payment methods for treatment expenditures are one of those factors influencing the length of stay of patients. In confirmation of the conceptualized element, a study by Yin et al. indicated that there is a significant relationship between the level of activity-based financing for service delivery and the inpatients’ length of stay in the hospital(Yin et al., 2013).

Cooperation with insurance for paying the health care expenditures is also one of the conceptualized elements. One of the desirable goals of any healthcare system is to provide appropriate mechanisms for cooperating with insurers to support households in demand for health services. In the confirmation of the component, the results of the study by Sepehri et al. demonstrated that the plan of compulsory insurance and the insurance plan for the poor will increase the length of stay of hospitalized patients(Sepehri et al., 2006).

The support of other public sector organizations from the provision of the healthcare services is also a priority. Among the factors influencing the length of stay of patients, it is noteworthy to mention the cooperation, and support of government organizations such as health insurance, armed forces, the Imam Khomeini relief committee and other insurance organizations which can affect the utilization and availability of the health care services for the people and consequently, influences on the length of stay. The results of a study by Vatankhah et al., (2004) proved that the length of stay of patients varies with the types of health insurance, so that patients under-coverage of the relief committee insurance had longer length of stay, and the reason may be the free treatment of patients by using this insurance. On the other hand, people without insurance had the minimum length of stay in order to avoid catastrophic payments due to the fact that they are forced to pay the healthcare expenditures individually (Vahidi et al., 2006). The culture of the target community toward the utilization of the healthcare services is also another conceptualized notion because health services are formed and shaped by cultures, traditions, payment mechanisms and patient expectations. Various studies have shown that the culture of individuals, their education, and their knowledge has had an important impact on the access of individuals to treatment services (Khayatan et al., 2011). Also, informing patients about the provided and offered healthcare services and their participation in decisions about their own treatment improves their health status and well-being and affects the inpatient’s length of stay. Schaffer stated that preoperative training reduces the length of stay (Sheaffer et al., 2018). On the other hand, based on the research evidence, among the socioeconomic factors, the financial resources of the patient and his/her type of job and occupation can be considered as an obstacle or facilitator (Rahimian and Besharat, 2010). A study by Arab et al. emphasized that there was a significant relationship between the average length of stay in the hospital and individuals’ occupation (Arab et al., 2010).

*Functional components of the clinical staff*

 The function and performance of the clinical staff include the following: the expertise and experience of the medical staff, the planning and effective decision making of clinical processes and procedures, the use of new and effective therapies, coordination and collaboration with other levels of service delivery, compliance with clinical guidelines, avoiding the supplier-induced demand and moral hazard, and communication with patients. The expertise and experience of the medical staff are one of those factors influencing the length of stay. By upgrading the experience and expertise of the clinical staff, the patients’ recovery will improve, and subsequently, the length of stay will reduce. Studies indicated that the expertise and experience of physicians and clinical staff affect the length of stay (Ravangard et al., 2010; Ghiyasvandian et al., 2013).

Effective planning and decision making of clinical processes are also important. In confirming this component, a study by Joy at al. revealed the impact of decision making on the effectiveness of the service delivery and treatment (Choy et al., 2007). The utilization of new and effective therapies not only reduces the length of stay but also, improves the patient satisfaction. Xiao et al., (1997) has considered the effectiveness and efficiency of treatments as an effective factor in the length of stay of patients in Australian hospitals.

Coordination and collaboration with other levels of service delivery affect the efficiency of service delivery and reduce the unnecessary and prolonged inpatients’ length of stay. In acknowledgment of this conceptual component, Wee and Hopman, (2005) Pointed out that the variety of procedures and processes in different parts of the hospital, the quality and quantity of communication and inter-sectoral collaborations, including the link between admission and discharge, and support of units are among the influential factors on the length of stay, which has been proved in various studies.

Observing clinical guidelines is an important factor in reducing the unnecessary and prolonged length of stay. In confirmation of this conceptualized component, the study of Deister et al., (2017) suggested that administrative delays in admission increase the length of stay in a hospital.

Avoiding the supplier-induced demand reduces the unnecessary expenses and, on the other hand, increases the capacity for admitting actual patients. This result is consistent with the study by lux et al.(Lux et al., 2011). Communication with patients is also an important component in determining the length of stay. A study by Baggs et al. showed that effective communication between the patient and the caregiver leads to a reduction in the length of stay in the hospital(Baggs and Ryan, 1990).

*Components of Hospital Service delivery*

The components of health service delivery include the structure and capacity of health services provision, the type of provided health care services, the treatment equipment and facilities, the process of admission and discharge, the status of financial and performance indicators, the quantity and quality of clinical and non-clinical health services, observance of the patient rights charter and the patient safety culture, physical environment and amenities and respecting the rules governing the health care system. The structure and capacity of providing health care is an important factor in responding to needs, which in turn has an impact on the length of stay. Jama Minana et al. also considered the hospital’s capacity an influencing factor on the patients’ length of stay in their study(Minana et al., 2017). The type of provided health care services is also dominant and should be based on the needs and opinions of the patients in the healthcare organizations. In 2017, Junko Niimura et al. stated that there was a significant relationship between the type of services and length of stay of inpatients in Japan, they also demonstrated that providing home-care services can also reduce the length of stay in the hospital(Niimura et al., 2017). The equipment and facilities are also among those influencing factors in reducing the unnecessary and prolonged length of stays of patients. The hospital should also evaluate the quality of the provided services continuously, in order to determine and guarantee the existence of sufficient required equipment and materials in the units. A recent study by Ameri et al. indicated that the lack of sufficient diagnostic facilities or lack of adequate training for patients after discharge can lead to patient re-admission and longer length of stay (Ameri et al., 2015).

The sequence of admission, hospitalization, and discharge is also important and, any disruption in these processes will lead to the unnecessary and prolonged stay in the hospital. Falana et al., (2018) indicated that unnecessary diagnostic tests for the diagnosis and follow-up of the treatment increase the length of stay. The status of financial and operational or performance indicators is also an important factor. What can be deduced from similar studies is that performance indicators are representatives of the efficacy, effectiveness and productivity of an educational and therapeutic hospital, each of them can have a two-way effect on the length of stay of patients. The quality and quantity of clinical and non-clinical care services are also paramount, and if treatment procedures are not performed or are provided with poor quality, it will cause damage and, as a result, recursion, re-admission and prolonged length of hospitalization. García-Romero et al., (2017) proved that the quality improvement in clinical services and promotion of the medical research can reduce the length of stay in a hospital.

In order to ensure the quality of healthcare services, observance of the standards of medical ethics and patients ‘rights in the provision of health services is inevitable. Increasing the observance of patients’ rights will improve the quality of health care services. In this regard, in the study of Nasiri-Pour et al., a significant relationship was found between patient safety and ethics and the length of stay as a performance indicator of the hospital (Nasiripour and Jafari, 2016).

Also, the physical environment can be a good basis for approaching the medical standards and gaining the satisfaction of staff and patients. Compliance with the rules governing the provision of healthcare services has also had an impact on reducing the length of unnecessary stay in the hospital and has improved the effectiveness of the patient treatment. In confirmation of this conceptualized component, a study by Ameri et al., (2015) demonstrated that non-compliance with therapeutic protocols and guidelines leads to patients re-admission. Also, Minana et al., (2017)indicated that there was a significant relationship between the careful implementation of health care policies and the length of stay.

In conclusion, the current study has conceptualized the influencing dimensions and factors on inpatients’ length of stay in the therapeutic centers of Iran. At last, the structural model consisted of 4 dimensions and 29 factors influencing the length of stay of hospitalized patients. According to the statistical analysis, the relationship between the inpatients length of stay and these four dimensions and their components was significant and on the other hand, by considering the importance and priority among these four (patients’ conditions, background and underlying components, hospital service delivery, the clinical staff performance), the patients’ condition has the most influence on the length of stay of patients in medical centers of the country. The proposed structural model has also approved in the form of a general structure of the components. Therefore, this model can be used as an appropriate tool for assessing the importance of different factors affecting the length of stay of patients in Iranian health care centers in order to make effective decisions at the political and executive levels.
